# Dual-beam X-ray nano-holotomography

**DOI:** 10.1107/S1600577524003801

**Published:** 2024-06-25

**Authors:** Silja Flenner, Adam Kubec, Christian David, Imke Greving, Johannes Hagemann

**Affiliations:** a Helmholtz-Zentrum Hereon, Max-Planck-Straße 1, 21502Geesthacht, Germany; b Paul Scherrer Institut Forschungsstrasse 111 5232Villigen PSI Switzerland; c Center for X-ray and Nano Science – CXNS Deutsches Elektronen-Synchrotron – DESY Notkestraße 85 22607Hamburg Germany; d Helmholtz Imaging Platform Deutsches Elektronen-Synchrotron DESY Notkestraße 85 22607Hamburg Germany; Tohoku University, Japan

**Keywords:** multibeams, holography, nanotomography, dual beam configuration, holotomography

## Abstract

Nanotomography with hard X-rays is a widely used technique for high-resolution imaging, providing insights into the structure and composition of various materials. In recent years, tomographic approaches based on simultaneous illuminations of the same sample region from different angles by multiple beams have been developed at micrometre image resolution. Transferring these techniques to the nanoscale is challenging due to the loss in photon flux by focusing the X-ray beam. We present an approach for multi-beam nanotomography using a dual-beam Fresnel zone plate (dFZP) in a near-field holography setup. The dFZP generates two nano-focused beams that overlap in the sample plane, enabling the simultaneous acquisition of two projections from slightly different angles. This first proof-of-principle implementation of the dual-beam setup allows for the efficient removal of ring artifacts and noise using machine-learning approaches. The results open new possibilities for full-field multi-beam nanotomography and pave the way for future advancements in fast holotomography and artifact-reduction techniques.

## Abstract 2

The first implementation of full-field nano-holotomography in a dual-beam configuration is presented.

## 1. Introduction

Nanotomography is a widely used, destruction-free technique for high-resolution imaging of a wide range of materials, from biological specimens to inorganic materials, allowing researchers to study the structure and composition in 3D. This knowledge is relevant from large metre-sized objects down to the nanoscale where, for example, the porosity of a material influences the overall function of macroscopic objects, *e.g.* the activity of a catalyst (Alizadehfanaloo *et al.*, 2021 ▸) or the capacity of a battery (Zhou *et al.*, 2020 ▸).

A common imaging technique to obtain this knowledge is near-field holography (NFH) (Cloetens *et al.*, 1999 ▸). The sample is magnified by a divergent beam which is achieved by placing focusing X-ray optics in front of the sample. In this geometry, the maximum achievable resolution is given by the size of the focal spot. The contrast in the measurement of NFH is achieved by the propagation of the wavefield behind the sample in free space. Therefore, an additional phase-retrieval step is required to obtain the actual phase image of the sample. Usually, holographic projections are recorded at several propagation distances in order to retrieve the phase signal. In recent years however, iterative phase-retrieval approaches using only a single propagation distance (Hagemann *et al.*, 2018 ▸; Wittwer *et al.*, 2022 ▸) have been pushed forward which can compete with, or even outperform, standard multi-distance phase retrieval such as contrast-transfer-function-based techniques. Therefore, NFH offers the possibility to acquire full-field projections with only one exposure which allows for time resolved nano-scale imaging (Hagemann *et al.*, 2021 ▸).

It is common for the projections in full-field imaging to be acquired sequentially, *i.e.* one at a time.

Breaking this scheme is often motivated by the need for a faster tomogram acquisition, *e.g.* for fast time-dependent processes (Ruhlandt *et al.*, 2018 ▸; Meyer *et al.*, 2021 ▸; Rack *et al.*, 2014 ▸; Husband *et al.*, 2022 ▸).

In recent years, approaches have been developed which simultaneously illuminate the sample from different angles and thus obtain more than one projection per single exposure (Villanueva-Perez *et al.*, 2018 ▸; Voegeli *et al.*, 2020 ▸; Hoshino *et al.*, 2011 ▸; Bellucci *et al.*, 2023 ▸). This decreases the number of exposures needed to record a tomogram. So far, optical elements have been used to create two (Hoshino *et al.*, 2011 ▸; Duarte *et al.*, 2019 ▸) or multiple (Voegeli *et al.*, 2020 ▸; Sowa & Korecki, 2020 ▸) beams which overlap in the sample plane. Sub-micrometre resolution in a multi-beam setting has only been demonstrated in the soft X-ray regime (Duarte *et al.*, 2019 ▸) for a single projection using coherent diffractive imaging (Miao *et al.*, 1999 ▸). In the hard X-ray regime, multi-beam approaches for full-field techniques currently exist only in the micrometre range as they do not make use of any additional X-ray optics for magnification. The main challenge for multi-projection imaging at hard X-ray energies is to reach large deflection angles between the beams. This can be achieved, for example, via additional mirrors or a pair of multi-layer Laue lenses (Maser *et al.*, 2004 ▸; Kubec *et al.*, 2018 ▸; Flenner *et al.*, 2022*b* ▸). Though these are potential solutions to achieve large deflection angles, they require severe alterations in the setup since additional degrees of freedom are needed to align these optics.

In this paper, we use a specially designed dual-beam Fresnel zone plate to focus the incoming X-rays into two beams. We demonstrate the extension of full-field multi-beam imaging to the nanoscale for hard X-rays using two nano-focused beams in a magnified near-field holography setting. These beams form an overlap region at a certain distance behind the focus to create a sufficiently large field of view (FOV). The detector distance is chosen such that the two beams are separated in the detector plane. This multi-beam implementation does not yield large deflection angles, but it is straight forward to implement in the existing setup and does not require additional degrees of freedom to align. By acquiring two projections simultaneously, the setup can be used to efficiently remove noise and ring artifacts in the tomograms via a machine-learning approach.

## 2. Experimental setting

**Figure 1:**
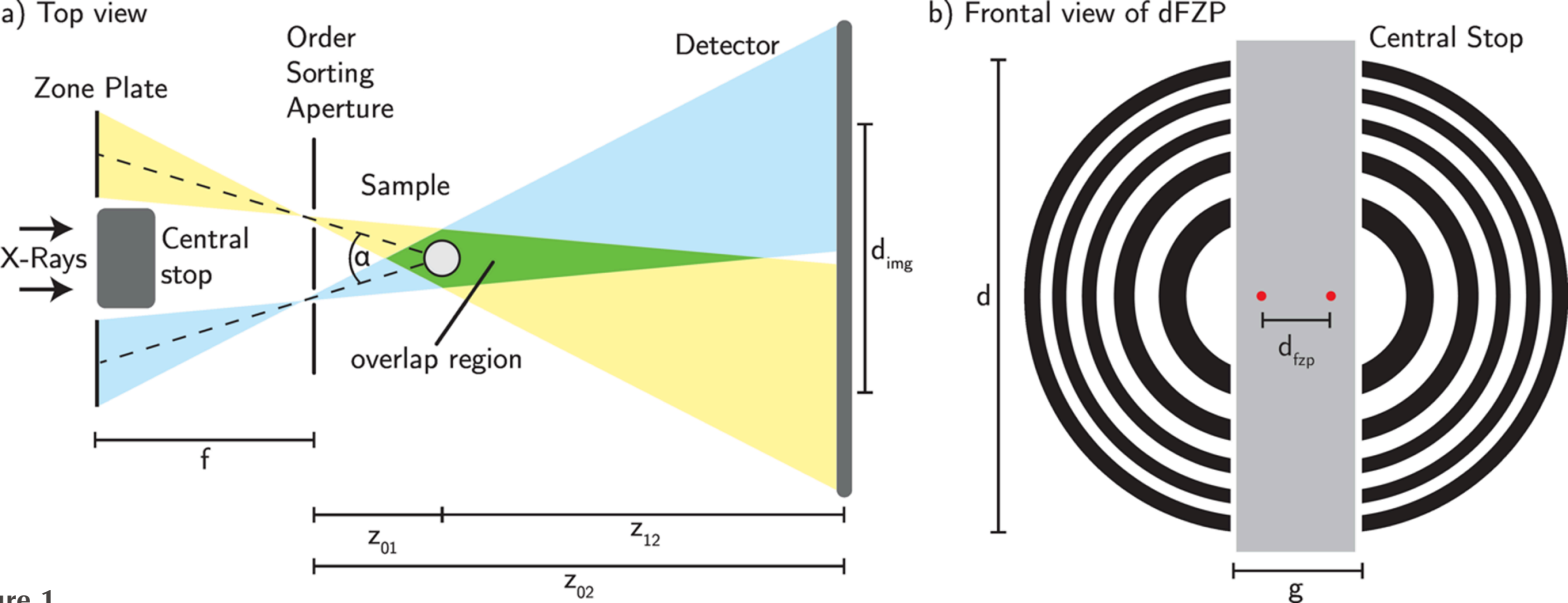
Multi-beam setup at P05. (*a*) Top view of the beam path. The beams overlap in the sample region. (*b*) Frontal view on the dual-beam FZP. The two centers of the parent FZPs are indicated by red dots. A summary of the experimental parameters is given in Table 1 ▸.

The experiment was implemented at the nano-imaging setup of the beamline P05 at PETRA III (DESY, Hamburg) operated by Helmholtz–Zentrum Hereon. The X-ray beam is monochromized with a channel-cut Si(111) double-crystal monochromator (DCM) to an energy of 11 keV. The nanotomography setup can be configured in a near-field holography mode using a Fresnel zone plate (FZP) as the focusing optics (Flenner *et al.*, 2020*a* ▸). In order to obtain the multiple-beam geometry, a specifically designed dual-beam FZP (dFZP) is used instead of a standard FZP. The integration of this dFZP was straightforward, since no alteration of the setup was necessary. Fig. 1 ▸(*a*) shows a schematic top view of the setup. The design of the dFZP consists of two halves of single FZPs. Their centers are separated to create an overlapping region in the sample plane, as shown in Fig. 1 ▸(*b*). The respective optical axes of the individual parts of the zone plate have a distance from the center axis of 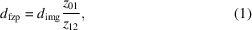
where *z*_01_ and *z*_12_ are the sample-to-focus and sample-to-detector distances and *d*_img_ is the distance of the two images at the detector. Note that *d*_img_ has to be chosen in such a way that the images do not overlap on the detector. The separation of the FZPs is adapted to the target FOV = 50 µm, corresponding to an effective pixel size Δ*x* = 197 nm. This defines the defocus distance *z*_01_, in which both beams overlap: 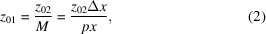
where *M* is the magnification, *px* the physical pixel size of the detector and *z*_02_ is the focus-to-detector distance. The design parameters of the dFZP are listed in Table 1 ▸. The resolution of the setup is determined by the theoretical resolution of the dFZP, but also the coherence properties of the source affect the final resolution. At an energy of 11 keV, the transverse coherence length is 105 µm in the horizontal direction and 600 µm in the vertical direction (Flenner *et al.*, 2020*a* ▸). This impact of partial coherence needs to be considered when describing the size of the focal spots created by the dFZP. This involves the convolution of the geometric image of the source with the point spread function of the optics. Taking this into account, we expect a focal spot size of 231 nm. The manufacturing process of the zone plate is done using electron beam lithography and electroplating (Gorelick *et al.*, 2011 ▸). The theoretical achievable resolution is approximately 100 nm and is limited by the numerical aperture of the optics. A central stop of 100 µm-thick tungsten is placed between both parts of the dFZP in order to block the direct beam. The order-sorting apertures are mounted in the focal plane to block the direct beam and higher diffraction orders of the dFZP.

An Si-based photon counting Xspectrum LAMBDA 750k with a pixel size of 55 µm is used as the detector (Flenner *et al.*, 2023 ▸). This detector consists of 12 individual chips of 256 pixels × 256 pixels arranged on a 2 × 6 grid. As the gaps between the chips are covered by larger pixels which are split into three pixels in the detector software, the final size of LAMBDA is 1556 pixel × 516 pixels or 85.57 mm × 28.38 mm. The large area of the detector allows to simultaneously capture the image of both beams. To achieve high resolutions with the large pixels of the LAMBDA detector, a high magnification in the X-ray regime is realized by the geometry of the setup. Therefore, the dFZP was designed such that the two beams overlap 70.66 mm behind the focus.

The experimental distances differ slightly from the design values (Table 2 ▸). In the actual experiment, the focus-to-sample distance *z*_01_ was decreased such that a higher magnification was achieved. Given the sample-to-detector distance *z*_12_ = 20 735 mm, this corresponds to an effective pixel size of 133 nm.

## 3. Data acquisition and processing

A spider attachment hair was used as a test object for a tomographic scan (Schaber *et al.*, 2019 ▸; Flenner *et al.*, 2020*b* ▸). These hairs are located on the feet of spiders, enabling them to walk upside-down. It typically has a diameter of 5 µm to 15 µm and fine attachment elements with a diameter of 100 nm to 300 nm. As it is also a very low-absorbing sample, it is a perfect test sample for phase-contrast methods at the nanoscale (Flenner *et al.*, 2020*a* ▸). The spider attachment hair was placed in the overlap area behind the dFZP, and subsequently rotated to measure a set of 900 projections in the range 0° to 180°, corresponding to 1800 tomographic projections in the case of our dFZP illumination.

The angle α between the beams was measured with a flat silicon substrate. Its surface was aligned parallel with one of the beams by minimizing its width, then it was tilted so that the image became smallest in the other beam. The measurement was done with a step width of 0.01° (0.17 mrad). This way we found an angle α between the two beams of 1.22 mrad ± 0.17 mrad. Taking into account the large measurement error, the measured value is in good agreement with the design value of 1.14 mrad.

The data processing involves several steps. First, the acquired projections need to be flat-field corrected. The flat-field variation was extracted from 120 empty beam images, *i.e.* images of the illumination without a sample in the beam. These images were used as input for the principal component analysis from which 40 components were extracted and used to calculate the best-fitting flat field. Due to the very noisy border regions of the flat-field corrected ‘compound’ hologram, the two images were reconstructed separately by applying a mask with Gaussian fading on the respective hologram.

In the second step, the phase retrieval is performed on the flat-field corrected data. Here, an iterative projection-based phase-retrieval process in a refractive representation was used, as described by Wittwer *et al.* (2022 ▸). Being a pure phase object, the spider hair is straightforward to reconstruct as the absorption can be set to zero. Certain constraints were applied to the data: on the one hand, the measured X-ray intensity is used as a data constraint; on the other hand, the projected phase shift of the sample is restricted to the range [−∞, 0]. As an additional constraint, a self-refining support was applied starting in iteration 70. First, a threshold on the current iterate on the modulus of the reconstructed phases of 0.03 rad is applied, followed by an erosion with a diameter of 3 pixels to remove clusters of stray pixels. Afterwards, eroded outlines are restored with a 10 pixel-diameter dilatation. This yields the support for the following five iterations. The reconstruction result is obtained after 850 iterations. The reconstruction was implemented in the *HoloTomoToolbox* (Lohse *et al.*, 2020*a* ▸,*b* ▸). The described phase-retrieval scheme is applied to all projections of the tomographic dataset.

In a third step, these data are used to perform the tomographic reconstruction. Prior to the reconstruction, the projections are aligned to each other using an iterative re-projection algorithm, also available in the *HoloTomoToolbox*. The tomographic reconstruction was then performed using the *gridrec* algorithm with a Shepp–Logan filter implemented in *Tomopy* (Gürsoy *et al.*, 2014 ▸).

## 4. Results

**Figure 2:**
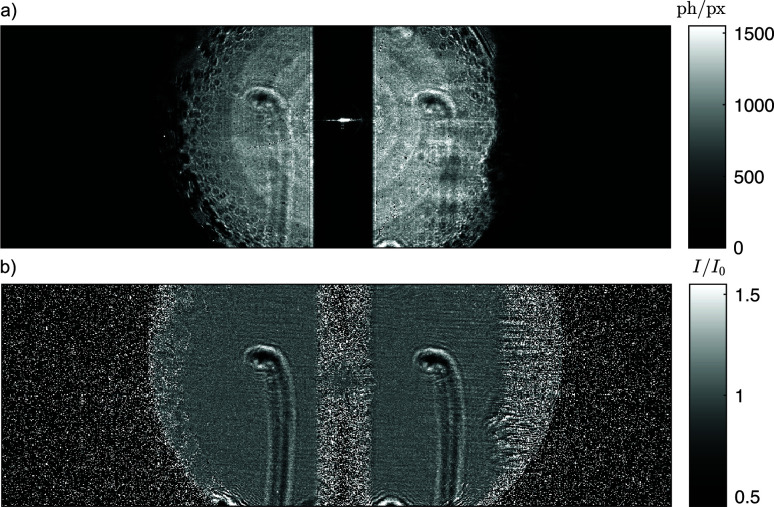
Example for holographic data acquired with the dual-beam FZP. The images show full frames of the LAMBDA detector. (*a*) Raw image of the two holograms on the detector. Between these, the primary beam leaks through the central stop, but the intensity of this peak is below the saturation rate of LAMBDA. The black dots on the outer edge of the illuminated areas are caused by imperfections in the dFZP. (*b*) Flat-field corrected holograms obtained via dynamic flat-field correction.

**Figure 3:**
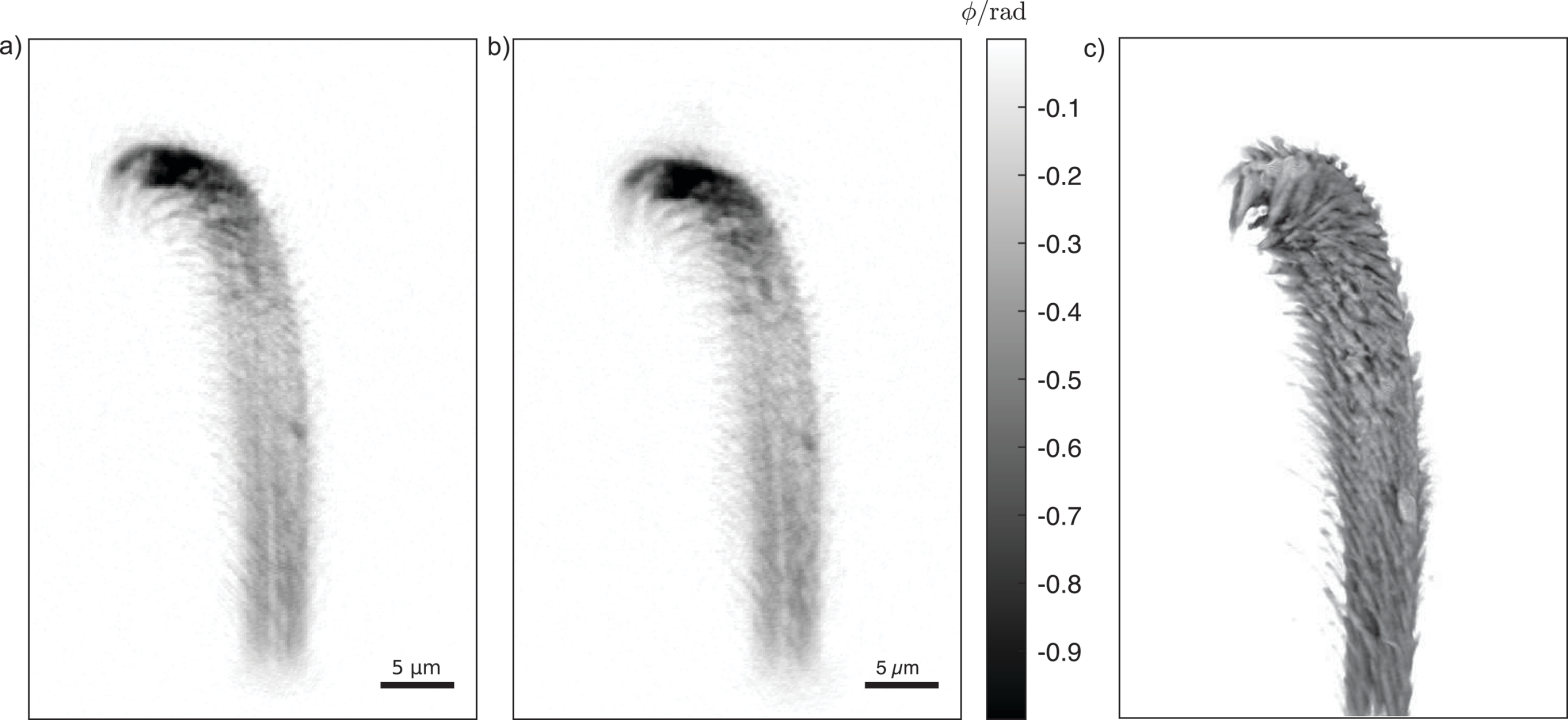
Phase reconstructions of (*a*) left illumination and (*b*) right illumination. The two projections are recorded in a single exposure with a difference in projection angle of 1.22 mrad. (*c*) 3D rendering of the denoised tomographic reconstruction.

The multi-beam near-field holography method was successfully implemented at the nanotomography setup at P05. Fig. 2 ▸(*a*) shows exemplary raw data from LAMBDA. The image shows the left and right first-order beam of the dFZP. The spider hair is visible on both sides at the same time, illuminated from a slightly different angle. The dark spots on the outer rim of the illuminated area stem from imperfections in the manufacturing of the dFZP. In between the focused beams, the attenuated primary beam hits the detector. These artifacts can be removed via principal-component-based flat-field synthesis (van Nieuwenhove *et al.*, 2015 ▸; Hagemann *et al.*, 2021 ▸), as shown in the flat-field corrected hologram in Fig. 2 ▸(*b*). The exposure time was set to 2 s which results in an average flux per pixel of 500 photons pixel^−1^ s^−1^. The efficiency of this proof-of-principle dFZP is comparatively low. For comparison, a standard gold FZP (300 µm diameter) of better quality yields 2000 photons pixel^−1^ s^−1^ (Flenner *et al.*, 2023 ▸). However, the dFZP can, in principle, also be manufactured with the same structure height as conventional FZPs, yielding a comparable efficiency. Fig. 3 ▸ shows the phase reconstructions obtained from the left [Fig. 3 ▸(*a*)] and right [Fig. 3 ▸(*b*)] hologram from the same compound hologram.

By recording a single tomography scan with the dual-beam setup, two sets of projections are acquired simultaneously which are separated by an angle of 1.22 mrad (0.07°). In a standard tomography scan, the angle between neighboring projections depends on the number of pixels, or in the case of region of interest, scans on the size of the object. The Crowther criterion describes the number of projections needed in a tomogram (*n*_proj_) and can be formulated as (Crowther *et al.*, 1970 ▸; Jacobsen, 2018 ▸): *n*_proj_ = π(*D*/*d*), where *D* denotes the size of the object under study and *d* the resolution. In the case of objects fitting in the FOV and resolutions of two pixels, this simplifies to *n*_proj_ = π(*n*_pix_/2), where *n*_pix_ denotes the horizontal number of pixels of the detector. By combining the projections of both sides into a single tomographic stack, undersampling can be avoided. Since in our case only 220 pixels of the detector are used, the Crowther criterion is already fulfilled at an angular separation of approximately 8.7 mrad (0.5°). Therefore, the combination of the two tomograms does not reduce the total number of exposures needed in this particular case.

Currently, the setup is limited by the manufacturing process of the optics. The deflection angle mainly depends on (i) the outer most zone width of the FZP and (ii) the ratio of the written aperture to the diameter of the parent FZP. The first one (i) is limited by the manufacturing: high-efficiency high-resolution FZP for hard X-rays are still challenging to manufacture. The outer most zone width can therefore only be reduced to around 30 nm for 11 keV optics for regular FZP while maintaining a reasonable efficiency. Smaller zone widths are possible but only with lower structure heights (Chao *et al.*, 2009 ▸; Mohacsi *et al.*, 2017 ▸; Rösner *et al.*, 2018 ▸). The second one (ii) on the other hand worsens the resolution, because fewer structures contribute to the focusing. In addition, the coherence length has to be considered when selecting the size of the FZP (Flenner *et al.*, 2020*a* ▸). From these constraints, we estimated a maximum deflection angle of approximately 3.0 mrad (0.17°) at the 30 nm outer most zone width at the same effective pixel size and resolution. With the setup described here, it is not possible to add additional information to the measurement due to the Crowther criterion. For samples exceeding the FOV, *i.e.* when more projections are required to fulfill the Crowther criterion, this approach can be used to decrease the acquisition time. However, with improving properties of X-ray optics, an increase in the possible deflection angle can be expected in the future. This can already be achieved by combining two crossed multi-layer lenses. In the future, improved lithography technologies will be able to achieve larger angles as well by allowing higher aspect ratios for smaller dimensions or by using higher orders of diffractive optics. Additionally, it would be possible to increase the angle by a combination of different approaches such as combining focusing refractive and diffractive optics and potentially reflective optics.

While improving the time resolution, a high image quality must also be maintained. Ring artifacts and noise are two common obstacles that obscure the image quality in X-ray tomography. These can be reduced using time-consuming scanning protocols or extensive post-processing (Boin & Haibel, 2006 ▸; Pelt & Parkinson, 2018 ▸; Münch *et al.*, 2009 ▸). Among these methods, machine-learning approaches have also been developed which offer a powerful alternative to conventional methods. However, usually only one, artifact or noise, can be reduced at the same time while risking the introduction of additional artifacts (Pelt & Parkinson, 2018 ▸; Prell *et al.*, 2009 ▸). Machine-learning denoising, for example, can significantly enhance artifacts like ring artifacts (Flenner *et al.*, 2022*a* ▸). On the other hand, ring removal often leads to less contrast and loss of spatial resolution (Prell *et al.*, 2009 ▸).

**Figure 4:**
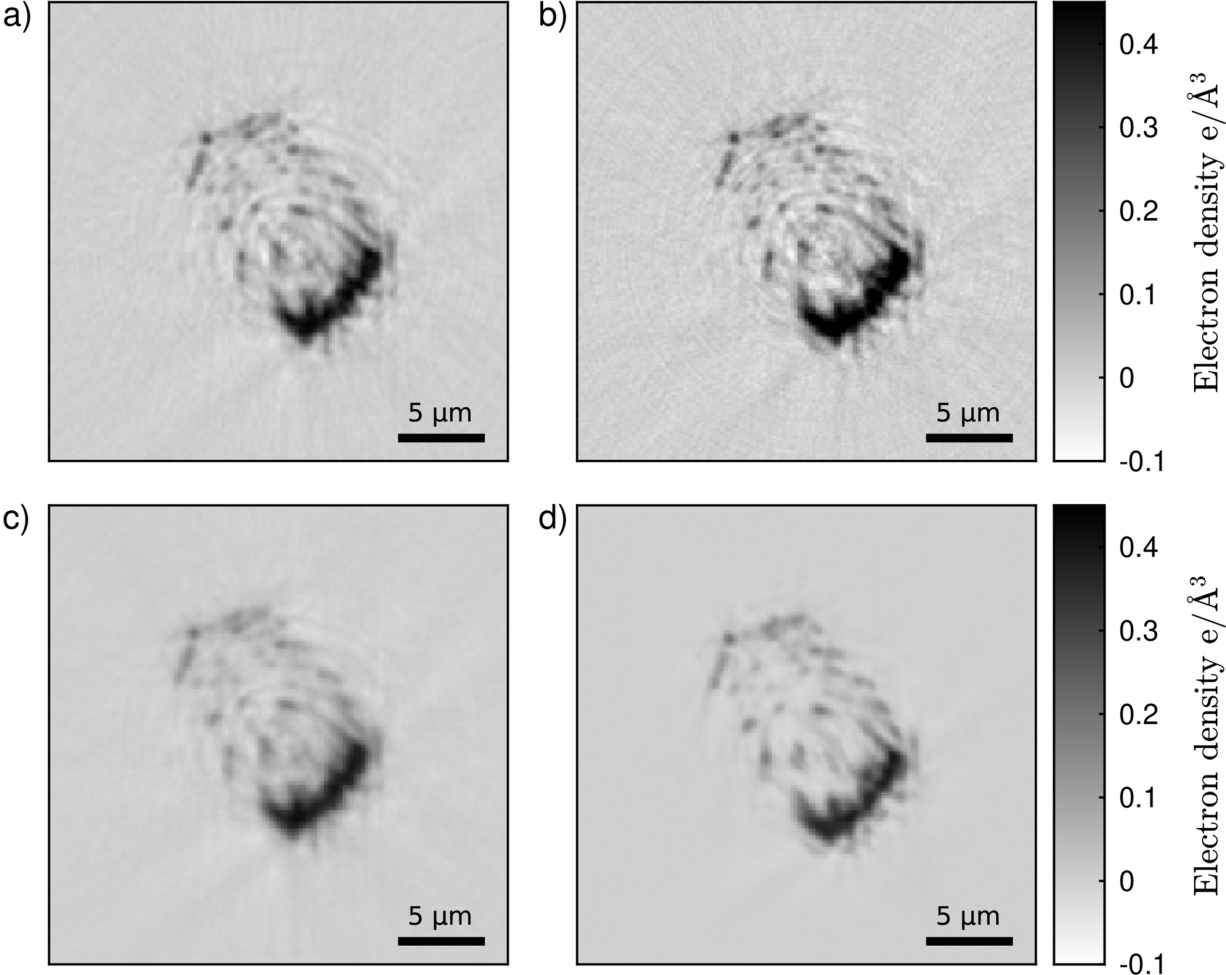
Reconstructed slices of the (*a*) left and (*b*) right projections of the spider attachment hair. The ring artifacts especially obscure the image quality. (*c*) Slice reconstructed by combining left and right projections. (*d*) Results from the machine-learning approach, applied to the tomogram of the right projections. Noise and ring artifacts are significantly reduced.

Here, the dual-beam setup offers the opportunity to maintain a high image quality and efficiently remove ring artifacts in the tomograms: in a simple approach, the projections are combined to reconstruct a single tomogram. Before reconstruction, the projections of both sides need to be aligned to be able to serve as input for the tomographic reconstruction. In this way, artifacts are averaged and therefore reduced significantly [Fig. 4 ▸(*c*)] compared with the single reconstructions [Figs. 4 ▸(*a*) and 4 ▸(*b*)], but some blurring is introduced due to imperfections of the alignment of the projections.

A more advanced and very powerful approach utilizes machine learning: the reconstructed tomographic volumes of both sides are aligned and used as the input and reference to train a convolutional neural network, as described by Flenner *et al.* (2022*a* ▸). As the noise, as well as possible defects and features on the detector are different on both sides, noise and ring artifacts can be efficiently removed at the same time. Fig. 4 ▸ shows slices of the two aligned tomograms used as the input for training [Figs. 4 ▸(*a*) and 4 ▸(*b*)], and the output after applying the trained network to the reconstruction of the right projections [Fig. 4 ▸(*d*)]. Noise and ring artifacts are successfully removed from the result, whereas in this case the image is less blurred due to imperfect alignment. Overall, this method leads to an improved image quality compared with the pure combination of both sides [Fig. 4 ▸(*c*)]. This noise and artifact-free image is key to enable 3D segmentation of the sample [Fig. 3 ▸(*c*)] and open doors towards a quantitative analysis.

## 5. Conclusions and outlook

We have demonstrated the first experimental implementation of a full-field nanotomography setup with two nanofocused beams that can achieve 133 nm voxel resolution. In this proof-of-concept experiment, we used an FZP design with two beam-forming elements (dFZP) with overlapping beams in the sample plane. The possible applications of this concept are versatile and bear large potential in the development of fast holotomography and for the elimination of noise and ring artifacts.

In the current study, both beams had the same optical properties. Extending the demonstrated principle using the additional information gained by simultaneous recording offers great potentials which can be exploited via different approaches: in NFH, a variation of defocus can yield additional information that can be used to stabilize the phase-retrieval process. This can be achieved by changing the focal length of one of the elements of the dFZP. Such a dFZP design allows us to measure holograms with two propagation distances simultaneously with a single exposure. In this case, the design of the FZPs can easily be adapted.

Another direction of development is to create more beams with larger deflection angles (Voegeli *et al.*, 2020 ▸; Villanueva-Perez *et al.*, 2018 ▸). This is not as straightforward, since it would require additional deflection of the beam with mirrors, which increases the complexity of the setup. Scanning-based imaging as far-field ptychography (Lyubomirskiy *et al.*, 2022 ▸) demonstrated great benefits from a multi-beam scanning scheme. Similarly, near-field holography can experience such gains of imaging speed which will pave the way towards tomographic movies (García-Moreno *et al.*, 2021 ▸) at sub-micrometre resolution.

## 6. Data availability

Data underlying the results presented in this paper are not publicly available at this time but may be obtained from the authors upon reasonable request.

## Figures and Tables

**Table 1:** Summary of dFZP parameters

Parameter	Value
Diameter of parent FZP, *d*	418 µm
Outermost zone width, Δ*r*_*n*_	61.4 nm
Theoretical spot size	197 nm
Focal length, *f*	228.3 mm
Angle between beams (design), α	1.14 mrad
Angle between beams (measured), α	1.22 mrad ± 0.17 mrad
Distance between FZP centers, *d*_FZP_	62 µm
Gap between FZPs, *g*	162.3 µm

**Table 2:** Summary of the imaging geometry

Parameter	Design value	Experiment
Defocus distance, *z*_01_	71.66 mm	50.29 mm
Sample-to-detector distance, *z*_12_	19928 mm	20735 mm
Detector distance, *z*_02_	20000 mm	20785 mm
Detector pixel size	55 µm	55 µm
Distances images at the detector, *d*_img_	17.3 mm	18.0 mm
Magnification	280×	413×
Effective pixel size, Δ*x*	197 nm	133 nm
Fresnel number	4.9 × 10^−3^	3.1 × 10^−3^
